# Development and validation study of the suicide screening questionnaire-observer rating (SSQ-OR)

**DOI:** 10.3389/fpsyt.2022.945051

**Published:** 2022-08-12

**Authors:** Young-Hwan Choi, Vidal Yook, Kyojin Yang, Yaehee Cho, Deok Hee Lee, Hwa Jung Lee, Dong Hun Lee, Hong Jin Jeon

**Affiliations:** ^1^Department of Psychiatry, Depression Center, Samsung Medical Center, Sungkyunkwan University School of Medicine, Seoul, South Korea; ^2^Department of Education, Traumatic Stress Center, Sungkyunkwan University College of Education, Seoul, South Korea; ^3^Department of Health Sciences and Technology, Samsung Advanced Institute for Health Sciences and Technology, Sungkyunkwan University, Seoul, South Korea

**Keywords:** suicide screening questionnaire-observer rating, suicide screening, risk assessment, psychometrics, reliability, validity

## Abstract

**Background:**

Observer rating scales are necessary to evaluate the risk of suicide because individuals at risk for suicide are often unwilling to seek help on their own. Reliability and validity were evaluated for the newly developed Suicide Screening Questionnaire-Observer Rating (SSQ-OR).

**Methods:**

Preliminary items were assessed by 251 experts online and 25 questions were selected. 328 individuals at high-risk and 661 controls from 12 Crisis Response Centers and 5 university counseling centers were recruited to complete SSQ-OR, Beck Scale for Suicide Ideation (BSSI) and Patient Health Questionnaire-9 (PHQ-9). In a 6 months follow-up, we reached out to 176 participants to ask whether they had experienced a suicidal thought, plan, or attempt since the baseline assessment. Cronbach's α, Mann-Whitney U test, Spearman's correlation, factor analyses, Receiver operating characteristic (ROC) analysis and logistic regression analysis were used to verify the SSQ-OR.

**Results:**

Structural validity was supported by a two-factor solution using exploratory and confirmatory factor analyses. Excellent model fit indices for the two-factor structure using exploratory factor analysis were confirmed (RMSEA = 0.033, TLI = 0.980, CFI = 0.983). The SSQ-OR demonstrated strong internal consistency. The concurrent validity based on the correlations with other self-reported indicators of suicidal potential–BSSI and PHQ-9– revealed substantial relationships. The high-risk group was effectively characterized by a cut-off point of 4, with a sensitivity of 0.73 and a specificity of 0.79. The SSQ-OR scores were significant predictors of suicidal thoughts and behaviors within 6 months.

**Conclusions:**

The SSQ-OR exhibits sound psychometric properties, and could be used as a complement to a self-report or clinical-administered scale to screen suicide risk comprehensively.

## Introduction

Suicide is a serious mental health concern, with approximately 800,000 suicide deaths reported annually worldwide (1). Similarly, suicide rates have substantially increased over the past years in South Korea. Suicide is the fifth leading cause of death, and a high mortality rate of 25.7 per 100,000 population in 2020 is reported (2). Despite extensive research over the past 50 years, predicting suicide risk is still limited and are not effectively implemented (3, 4). This underscores the need for developing an assessment tool that addresses limitations of current suicide risk assessment.

Most of the suicide risk assessment tools only focus on certain risk factors such as suicidal thoughts, suicidal behavior, or other risk factors (5, 6). However, previous literature have that suicidal thoughts and behaviors (STBs) are caused by a complex interaction of various proximal and distal factors (3, 7–9). It is necessary to include a broader context of risk factors based on relatively distal risk factors as well as STBs (10). Based on the results of previous suicide studies (3, 7, 9), when designing a tool to screen the risk of STBs, it is necessary to consider risk factors not sufficiently covered by current assessment tools such as the social factors, negative life events, and physical disorders.

In a recent systematic review of risk scales, the use of a single instrument are not recommended in clinical settings (11). Rather than relying on a single tool, there is a need to combine multiple risk assessment tools together to effectively measure suicide risk. Moreover, non-disclosure or denial of suicidal ideation is quite common among psychiatric patients and those who attempt and/or complete suicide (12–14). Since concealment is a risk factor for STBs (15, 16), it is likely that the evaluation using only self-reports or clinician interviews might have limitations. The observer rating complements the self-report or clinician-administered scale since it is based on direct observation of subjects' behavior in a daily environment. The observer rating method accounted for significant variance beyond self-report and revealed unique information relative to self or assessor evaluations in suicide behaviors (17, 18). In particular, suicide warning signs, revealed through psychological autopsy, allow observers to detect the imminent risk of STBs (19). Hence, it can be helpful to use an observer rating scale that complements the existing instruments when screening individuals with suicide risk.

To this end, the present study was undertaken to develop a more comprehensive, observer-rated screening tool for identifying suicide risk. The study aims to develop and validate a Suicide Screening Questionnaire-Observer Rating (SSQ-OR). We described the process of (1) investigating structural validity, (2) examining of the measure's concurrent validity, predictive validity, and internal consistency, and (3) setting the best cut-off score that discriminate between individuals with a high risk of suicide and those with at low risk.

## Methods

### Scale development

For scale development, preliminary questions were constructed through extensive research on existing suicidal risk scales, previous studies on suicide risk factors [e.g., Franklin et al., Rudd et al., O'Connor and Nock (3, 7, 8)], qualitative interviews with individuals who have a history of STBs and counselors, and the nationwide reports on suicide deaths [e.g., (20, 21)]. According to psychological autopsy results, warning signs were observed in more than 30% of suicide decedents, and these warning signs were reflected in the preliminary items (22). In order to explore the characteristics of high-risk groups for suicide that can be observed by the subject's families or acquaintances, counseling psychologists interviewed various groups at high risk of suicide and performed a qualitative study. Based on these findings, we generated a total of 64 preliminary items composed of 5 categories: suicidal behavior, mental health problems, temperament & personality, environmental factors, and warning signs.

Next, we evaluated the relevance and clarity of each question as well as the content validity. We conducted an online survey of 251 mental health professionals in the field of psychiatry, clinical psychology, counseling psychology, social welfare, education, or nursing, to evaluate the relevancy and clarity of each item. Based on the results of the first survey, a group of 17 professionals (i.e., psychiatrists, clinical psychologists, counseling psychologists) selected 39 questions and refined the wordings of each question. Then each professional rated the degree of adequacy and significance of the items on a five-point scale on (1) adequacy and (2) significance of the item (ranging from 1= ‘very inappropriate/not important' to 5= ‘very appropriate/important'). Finally, 25 items were selected after excluding those with low scores in adequacy or importance. Supplementary Appendix A presents the relevance and importance scores of the final 25 items rated by the 17 experts.

### Procedure

We recruited participants from 12 Crisis Response Centers and 5 university counseling centers across South Korea between October 2020 to March 2021. These centers recruited community-dwelling adults as well as visitors to the center. The Crisis Response Center operated by university hospitals provide case management services for those who visit the emergency room due to a suicide crisis. Inclusion criteria were being at least 18 years old and having the reading level of a middle school graduate. Participants were excluded if they had neurodegenerative disease, intellectual disability, clinically significant personality disorder, brain injury, and any other conditions that might interfere with participation. After a full explanation of this study, those who voluntarily signed the consent form were included. The compensation was provided for both participants and clinicians. All participants were interviewed in a one-on-one setting by trained clinicians of the centers and asked to complete a set of self-report questionnaires. The SSQ-OR requested a rating from the subject's families or acquaintances including counselor/social worker, spouse, friend/colleague, lover, and teacher/professor. Clinicians interviewed the participants from Crisis Response Centers 6 months later to establish whether there had been suicide ideation, plan, or attempt since the baseline assessment. This study was approved by the Institutional Review Board of the Samsung Medical Center (IRB No. SMC 2020-04-184).

### Participants

A total of 989 subjects participated in this study. We used the term ‘suicidal group' for those who had suicidal ideation and/or suicide attempt together as a high-risk group. We defined the suicidal group as those who thought about killing themselves but had not attempted suicide and/or those who had attempted suicide within 6 months and the control group as those who did not have suicidal ideation, suicidal plan, or suicidal attempt within 6 months. Among the total participants, 328 (33.16%) were recruited for the suicidal group and 661 (66.84%) for the control group. The information of observers who rated SSQ-OR is as follows: counselor/social worker 435 (43.98%), friend/colleague 160 (16.18%), family 142 (14.36%), teacher/professor 95 (9.61%), spouse 63 (6.37%), lover 51 (5.16%), and others 43 (4.35%). Out of all subjects, 176 (17.80%) were followed up through phone calls after 6 months to assess whether they have ever experienced STBs (i.e., suicidal ideation, suicide plan, and suicidal attempt). Among those, 157 participated in this follow-up interview and 19 did not answer.

### Measures

#### Suicide screening questionnaire-observer rating (SSQ-OR)

Based on the aforementioned procedures, the final version of the SSQ-OR consisted of 25 items. The SSQ-OR asks the informants, such as families or acquaintances, to rate the presence of observable behaviors of an individual over the last month on a binary scale (yes/no). The total score of SSQ-OR ranges from 0 to 25. The ‘do not know' response to each item is not included in the total score, and the range of the response rate of ‘do not know' (see Supplementary Appendix B) was 0.93 to 6.41% in the present study. Except for item 8 (“Has attempted suicide more than once so far”) and item 13 (“Says he/she wanted to die following a deceased family member, friend, pet or celebrity”), the response rates of ‘do not know' were <5%.

#### Beck scale for suicide ideation (BSSI)

To verify the concurrent validity of the SSQ-OR, we used the BSSI, the most frequently used measure of suicidal risk. BSSI developed by Beck, Steer (23) consists of 21 questions that measure suicidality and the severity thereof. Based on the participants' experience of the past weeks, a three-point Likert scale (0–2 points) was used. The psychometric properties of the BSSI have been established for Koreans (24, 25). The value of Cronbach's alpha for this study sample was 0.92.

#### Patient health questionnaire (PHQ-9)

The PHQ-9 is a nine items self-report measure used to assess the severity of depression. The items are based on the DSM-IV's diagnostic criteria for major depressive disorder. On each of the nine items, participants are asked to self-rate how often they have experienced the indicated symptoms of depression over the previous 2 weeks on a four-point Likert scale (0–3 points). The Korean version of PHQ-9 standardized by Han, Jo (26) and Kim and Lee (27) demonstrated adequate internal consistency and convergent validity. The Cronbach's alpha coefficient of the study was 0.91.

### Statistical analyses

The Statistical Package for Social Sciences 21 was used to examine descriptive characteristics, concurrent validity, internal consistency, corrected item-total correlation, Receiver Operating Characteristic curve (ROC) analysis, and predictive validity of the sample. Intergroup comparisons were conducted using the Mann–Whitney U-test. Correlation analyses were performed using Spearman's rank correlation analysis. The prevalence of the lowest scores, 0/25 (floor effect), and highest scores, 25/25 (ceiling effect), for the SSQ-OR were calculated. If more than 15% of the participants scored maximum or minimum scores, we considered these to be floor and ceiling effects, respectively. We adopted a series of analyses to evaluate its structural validity. Bartlett's test of sphericity and the Kaiser-Meyer-Olkin index (KMO) were performed to determine whether the data is suitable for factor analysis. In order to identify the number of factors to retain, Kaiser's criterion, Cattell's scree test, and parallel analysis based on Minimum Rank Factor Analysis (PA-MRFA) were applied using Factor 10.10.02. We randomly split the total sample into two separated samples using RANDBETWEEN function in Microsoft Office Excel 2016. Based on the two split samples, an exploratory factor analysis (EFA) and a confirmatory factor analysis (CFA) were conducted with geomin rotation using Mplus 7.0. Due to the ordinal nature of the SSQ-OR item responses, the SSQ-OR models were examined treating the items as categorical with the mean-and variance-adjusted weighted least squares estimator (WLSMV) (28, 29) which has been found to be robust to violations of normality Missing values were treated as pairwise missing by convention with this estimator. Assessment of the fit of each model was based on several indices. Since the χ2 statistic is highly sensitive to sample size (30), three fit indices were considered together: (1) the comparative fit index (CFI), (2) the Tucker Lewis index (TLI), and (3) the root mean square error of approximation (RMSEA). According to the criteria proposed in previous studies, RMSEA <0.05 and CFI and TLI > 0.95 was considered to be good (31). Also, binary logistic regression analyses were conducted to investigate whether the SSQ-SR total and sub-factor scores predicted STBs within 6 months. ROC analysis was done to evaluate the screening ability of SSQ-SQ to discriminate between suicidal and control groups. We investigated the best cut-off point with the maximal area under the curve (AUC) and sensitivity/specificity. AUC values of >0.9, 0.8–0.9, 0.7–0.8, 0.6–0.7, and 0.5–0.6 are regarded as excellent, very good, good, sufficient, and bad diagnostic accuracy, respectively (32).

## Results

### Demographic characteristics

Table 1 shows descriptive results of basic characteristics. All of the participants were between 18 and 68 years of age (Mean age = 26.94, *SD* = 9.34). The male-to-female ratio was 1:1.76. There was no significant difference in the total score of SSQ-OR across gender, U = 85,585.50, *ns*.

**Table 1:** Demographic characteristics of the study sample and scores of suicide screening questionnaire-observer rating (SSQ-OR).

**Characteristic**	**Suicidal group** ***(n* = 328*)***	**Control group** **(*n* = 661)**	**Total** **(*N* = 989)**
Age, mean (SD)	26.92 (9.79)	26.94 (9.12)	26.94 (9.34)
Female (*n*, %)	234 (71.34)	397 (60.06)	631 (63.80)
**Years of education received (*n*, %)**			
0–12	204 (62.20)	362 (54.77)	566 (57.23)
13–16	105 (32.01)	240 (36.31)	345 (34.88)
More than 16	19 (5.79)	59 (8.93)	78 (7.89)
**Region (** ***n*** **, %)**			
Urban cities	298 (90.85)	593 (89.71)	891 (90.09)
(including Seoul)			
Rural areas	30 (9.15)	67 (10.14)	97 (9.81)
**Marital status (** ***n*** **, %)**			
Single	262 (79.88)	536 (81.09)	798 (80.69)
Married	44 (13.41)	114 (17.25)	158 (15.98)
Others	22 (6.71)	11 (1.66)	33 (3.34)
**Occupation (** ***n*** **, %)**			
Employed/	83 (25.30)	249 (37.67)	332 (33.57)
Self-employed			
Student	161 (49.09)	352 (53.25)	513 (51.87)
Unemployed	65 (19.82)	38 (5.75)	103 (10.41)
Others	19 (5.79)	22 (3.3)	41 (4.15)
**Living arrangement (** ***n*** **, %)**			
Living alone	75 (22.87)	133 (20.12)	208 (21.03)
Others	253 (77.13)	528 (79.88)	781 (79.97)
**SSQ-OR**			
Total score	7.94 (5.55)	2.09 (3.12)	4.18 (5.01)^*^
Factor 1	4.55 (3.46)	1.02 (1.72)	2.28 (3.00)^*^
Factor 2	3.32 (2.34)	1.12 (1.60)	1.90 (2.17)^*^

* p < 0.001.

P-values of Mann–Whitney U test for difference between suicidal group and control group. Factor 1 = Suicide and mental health; Factor 2 = Social and environmental stress.

Among 989 adults, 328 (33.16%) were in the suicidal group, which showed suicidal ideation or/and suicidal attempt over the last 6 months. Age of this group ranged from 18–68 years old (Mean age = 26.92, SD = 9.79), and 71.34% of them were female. Out of all samples, 661 (66.83%) were assigned to the control group, which showed no suicidal ideation or/and suicidal attempt in the last 6 months. The participants in the control group were between 18 to 67 years of age (Mean age = 26.94, SD = 9.12), and 60.06% of them were female.

### Structural validity

#### Exploratory factor analysis (EFA)

In determining whether the data is suitable for factor analysis, Bartlett's test of sphericity and the Kaiser-Meyer-Olkin index (KMO) were computed. The Bartlett's test was significant [χ2(300) = 3751.0, *p* < 0.001] and the KMO index was 0.91, which was judged to be appropriate data for factor analysis.

To identify the number of factors to retain, the eigenvalues of the polycorrelation matrix were reviewed (see Supplementary Appendix C). Initially, the three-factor solution with eigenvalues more than one was found using the Kaiser criteria. However, the Kaiser criterion might overestimate the number of factors (33). Further analysis of the Scree test supported the single-factor solution in that the eigenvalues drop significantly from the first to the next. Finally, the results of the parallel analysis (see Supplementary Appendix D) demonstrated that the 95th percentile eigenvalue for the random data distribution was larger than the eigenvalue of the raw score distribution for a single-factor solution. Referring to these results, the range of the number of factors of SSQ-OR that can be explored was determined to be 1–2.

We explored the underlying structure of SSQ-OR using EFA. A good model fit was obtained for a single-factor solution [χ2(275) = 483.780, *p* < 0.001; RMSEA = 0.042, TLI = 0.968, CFI = 0.970] and a two-factor solution [χ2(251) = 368.152, *p* < 0.001; RMSEA = 0.033, TLI = 0.980, CFI = 0.983]. Supplementary Appendix E presents the standardized factor loadings for the two-factor model. Based on Thurstone's criteria (34), the two-factor solution had: (1) zero items with salient loadings (≥0.40) on more than one factor, (2) zero items with no salient loading on any factor, and (3) well-defined salient loading per factor (i.e., factor 1 had 15 items, factor 2 had 10 items). With regards to the interpretability of the resulting factor structures and Thurstone's criteria (34), the two-factor model is found to be the most suitable for SSQ-OR. These factors were: (1) Suicide and mental health, and (2) Social and environmental stress. It was found that the factor loading of item 11 exceeded the absolute value of 1. However, the standardized regression coefficient might exceed the absolute value of one depending on the correlation between the independent variables and the correlation between each independent variable and the dependent variable (35).

#### Confirmatory factor analysis (CFA)

To confirm structural validity, CFA was performed on the other split sample. Based on the EFA results, our hypothetical factor model contained two factors: (1) Suicide and mental health, and (2) Social and environmental stress. A hypothesized two-factor solution showed a good model fit [χ2(274) = 438.003, *p* < 0.001; RMSEA = 0.037, TLI = 0.972, CFI = 0.974]. The standardized factor loadings of the CFA model are illustrated in Supplementary Appendix E. All factor loadings of the two-factor model demonstrated salient loadings (≥0.40). Therefore, the result of CFA cross-validated the two-factor structure suggested in the finding of EFA.

### Concurrent validity

In order to examine the relationship between SSQ-OR and other suicide-related measures, we used self-reported scales (BSSI, PHQ-9). Spearman's correlations were assessed (Table 2). The SSQ-OR total and sub-factors were moderately correlated with BSSI and PHQ-9. Additionally, the correlation between the sub-factors of SSQ-OR was 0.778.

**Table 2:** Means, standard deviations, and scale inter correlations.

**Scale**	***M***	**SD**	**1**	**2**	**3**	**4**	**5**
1. SSQ-OR	4.18	5.01	1				
2. Factor 1 (Suicide and mental health)	2.28	3.00	0.936^**^	1			
3. Factor 2 (Social and environmental stress)	1.90	2.17	0.910^**^	0.778^**^	1		
4. BSSI	5.84	7.90	0.575^**^	0.580^**^	0.508^**^	1	
5. PHQ-9	6.54	6.40	0.558^**^	0.555^**^	0.520^**^	0.692^**^	1

SSQ-OR, Suicide Screening Questionnaire-Observer Rating; BSSI, Beck Scale for Suicide Ideation; PHQ-9, Patient Health Questionnaire.

** p < 0.01.

### Internal consistency

Coefficient alphas and corrected item-total correlations were computed for SSQ-OR. The Cronbach's alpha of the SSQ-OR total was 0.91. The Cronbach's alpha was 0.89 and 0.75 for Factor 1 and 2, respectively. As shown in Table 1, total and sub-factor scores revealed significant differences between suicidal and control groups. The Cronbach's alpha if-item-deleted coefficients ranged from 0.90 to 0.91. The corrected item-total correlations showed that all SSQ-OR items were significantly correlated with the SSQ-OR total scale score (Supplementary Appendix B). These values support the internal consistency of the SSQ-OR scores.

As for the floor and ceiling effects, the floor effect identified the percentage of individual results that corresponded to the theoretical minimum (0/25). 266 of 989 subjects (26.9%) scored zero so there was a floor effect. The absence of ceiling effect was observed, that is, none of the subjects scored the theoretical maximum (25/25).

### ROC analysis

**Figure 1:**
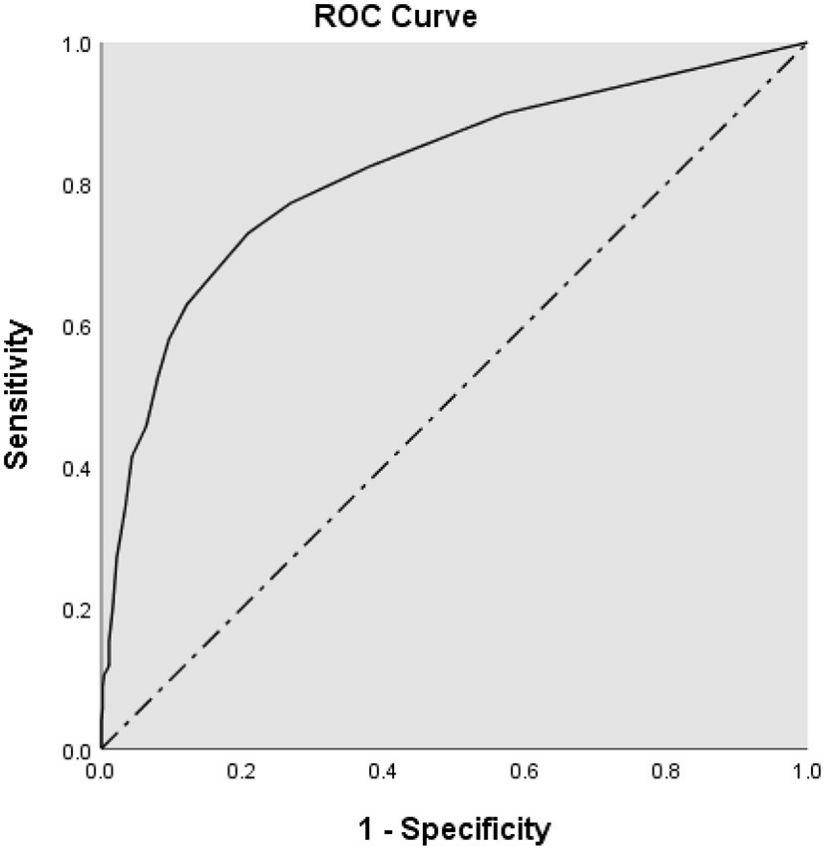
Receiver operating characteristic curve (ROC) analysis for the suicide screening questionnaire-observer rating (SSQ-OR).

Results of the ROC analysis are presented in Figure 1. In comparing the suicidal group with the control group, the AUC area was 0.817 (95% Confidence Interval: 0.786, 0.848) and the cut-off point of four provided the optimal balance between the sensitivity and specificity when considering Youden's J statistic (Table 3). The point decided by Youden's index is the farthest point from the diagonal line, where the sum of sensitivity and specificity can be maximal (20). At the cut-off point of four, the values of sensitivity, specificity, positive predictive value (PPV), and negative predictive value (NPV) are 73.0, 79.2, 66.1, and 84.2%, respectively. If the researcher wanted to maximize sensitivity for screening purpose, it would be possible to minimize false negatives by choosing the cut-off score of 3.

**Table 3:** Sensitivity, specificity for each cut-off score.

**Cut-off score**	**Sensitivity**	**Specificity**	**Youden's J**
0	1	0	0
1	0.899	0.430	0.329
2	0.824	0.621	0.445
3	0.772	0.733	0.505
**4**	**0.730**	**0.792**	**0.522**
5	0.671	0.843	0.514
6	0.629	0.879	0.508
7	0.580	0.904	0.484
8	0.524	0.921	0.445
⋮	⋮	⋮	⋮
22	0.007	1	0.007

The cut-point that maximizes Youden's index is bolded.

### Predictive validity

To investigate predictive validity, a total of nine logistic regression models were run with STBs (i.e., suicidal ideation, suicide plan, and suicidal attempt) as the outcome of interest for the subsample of those with 6 months follow-up data. Baseline SSQ-OR total and sub-factor scores were significant predictors of future STBs during the 6 months in adjusted analyses for gender and age (Table 4). The SSQ-OR total score was significantly associated with the outcome with the odds increasing by about 10% in all STBs. The sub-factor scores were also significantly associated with all future STBs: Factor 1 (Suicide and mental health) increased the odds ratio by about 10–20%, whereas Factor 2 (Social and environmental stress) increased the odds ratio by about 30–40%.

**Table 4:** Logistic regression models of the relationship between SSQ-OR and Suicide Ideation, suicide plan, or suicide attempt during 6 months follow-up (*n* = 157).

	**Suicide ideation**	**Suicide plan**	**Suicide attempt**
**Predictors**	**OR (95% CI)**	**OR (95% CI)**	**OR (95% CI)**
Total score	1.130 (1.068–1.197)^*^	1.173 (1.095–1.255)^*^	1.189 (1.098–1.288)^*^
Factor 1 (Suicide and mental health)	1.188 (1.096–1.287)^*^	1.254 (1.138–1.382)^*^	1.287 (1.146–1.445)^*^
Factor 2 (Social and environmental stress)	1.364 (1.154–1.612)^*^	1.465 (1.212–1.769)^*^	1.453 (1.182–1.787)^*^

Nine models adjusted for gender and age were run, each with the follow-up STBs (yes/no) as the outcome of interest. Includes 176 participants who were followed in 6 months and 19 did not answer. OR, odds ratio; CI, confidence interval.

* p < 0.001.

## Discussion

The present study evaluated the psychometric properties of the SSQ-OR (see Table 5 for the complete SSQ-OR questionnaire in English), the newly developed screening scale for suicide risk. It was found that the observer's 'don't know' ratio was generally <5%. That is, the SSQ-OR consisted of items that could be easily observed by acquaintances or family members of the subjects.

**Table 5:** Suicide screening questionnaire-observer rating; SSQ-OR.

	**Content**	**Yes**	**No**	**Do not know**
1	Rarely meets anyone and spends most of the day alone			
2	Suffers from conflicts with family members such as parents, children, or siblings			
3	Suffers from conflict, divorce, or breakup with a lover or spouse			
4	Depreciates or regards him/herself as pathetic			
5	Impulsively does something dangerous or regrettable (e.g., drunken driving, violence, binge eating, impulsive consumption, severe argument with people around)			
6	Says that he/she will die when emotions get intense			
7	Has planned a place, time, method for suicide (e.g., checking out a place or buying a tool, etc.)			
8	Has attempted suicide more than once so far			
9	Says that death is the only way to solve the current problems			
10	Talks about suicide or death			
11	Suddenly organizes the surroundings (e.g., property arrangement, personal affair arrangement, efforts to improve relationships)			
12	Says that people around him/her would be better off if he/she dies or disappears			
13	Says that he/she wanted to die following a deceased family member, friend, pet or celebrity			
14	Looks depressed or lethargic almost every day			
15	Has hurt him/herself to the extent of leaving a scar			
16	Continues drinking even though drinking causes serious problems (e.g., deterioration of health, violence/abusive language, interpersonal conflicts)			
17	Looks very anxious and nervous			
18	Has severe mood swings			
19	Is being suspicious of other people's intention and/or thinks others are doing him/her harm			
20	Complains about sleep problems (e.g., not being able to fall asleep easily, waking up in the middle of the night, change in sleeping hours)			
21	Suffers from a failure (e.g., job, promotion, business, academic failure, etc.)			
22	Suffers from the financial distress (e.g., debt, poverty, bankruptcy, etc.)			
23	Has suffered since experiencing physical, verbal or sexual violence			
24	Suffers from being unemployed			
25	Suffers from unfair treatment or insult			

Below are some of the things that people can experience from time to time. Please read the following questions carefully and mark the one that applies to the subject based on his/her subjective experiences over the last month.

e.g., If you are asked about a specific situation or event, such as “suffers from the death of a loved one” or “suffers from unfair treatment or insult,” please mark YES if the subject has been still suffering even if the episode had occurred before 1 month.

The structural validity was supported by a two-factor model using a series of EFA and CFA and showed good fit indices overall. Based on the loaded items of each factor, the two sub-factors indicated (1) Suicide and mental health and (2) Social and environmental stress, respectively. While the existing suicide risk measurements mainly focused on STBs or psychological factors related to STBs (5, 6, 36), the SSQ-OR also evaluated social and environmental stress corresponding to major risk factors (3). Suicide risk is a synergistic relationship between a number of intra- and inter-individual factors (37). Our findings were also consistent with several studies suggesting that suicide risk can be divided broadly into two categories: personal and social factors (38, 39). The total and sub-factor scores of the suicidal group were higher than those of the non-suicidal group, which demonstrated the content validity.

To investigate concurrent validity, we included the self-rated BSSI and PHQ-9 as measures of suicidal ideation and depression, respectively. It was found that moderate correlations between SSQ-OR total and suicidal ideation and depression scores. This is quite remarkable because the subject's acquaintances or families were not trained in the assessment and were simply instructed to score the SSQ-OR based on their observations in daily life. The present findings suggest a good convergence between self-reports and observer ratings. This was consistent with previous literature on the relationship between suicide risk and STBs. First, suicidal ideation is considered to be the starting point of STBs and is known to be a significant predictor of suicide attempts (40, 41). The severity of suicidal ideation is a major risk factor in predicting suicide leading to actual death. In particular, serious suicide attempters have previously reported suicidal thoughts lasting weeks to months, (42) and it was found that the probability of leading to a suicide attempt within 1 year after the accident occurred increased by 15–20% by countries (9). Second, depression is among the most commonly cited risk factors for STBs. Depression has been reported to be likely associated with the development of suicidal desire, and one of the prominent suicide theories, the Interpersonal Theory of Suicide, suggests that depression interacts with hopelessness and develops into STBs (43). Psychological autopsy studies have also repeatedly reported that depression is the most common psychiatric disorder among suicide deaths (22, 44). Consistent with theories and autopsy studies, a recent meta-analysis of longitudinal studies found that depression significantly confers future suicide ideation, attempt, and death (45). Therefore, concurrent validity was confirmed in that SSQ-OR showed a significant correlation with depression and suicidal ideation.

The SSQ-OR appeared to have strong internal consistency for total items and the sub-factors, and the item-total correlations were found to be favorable. Whereas no ceiling effects were found for the SSQ-OR, the floor effect was detected. This means that it could be hard to differentiate among the many individuals at a low level. It is possible that the binary scale (yes/no) to solicit observance of the subject's behavior might have led to a higher likelihood of floor effect. However, unlike other Likert scales, the developed tool was rated by the subject's informant and the binary scale seems appropriate. This is because it is difficult to respond on a Likert scale to behaviors observed by others, as well as the risk of distorting the responses of the actual subject.

A ROC analysis was performed to identify the screening performance of SSQ-OR. The AUC of 0.817 suggested a very good accuracy (32). It was slightly higher than the self-reported screening version of Columbia-Suicide Severity Rating Scale (C-SSRS) with an AUC value of 0.72 (46). A cut-off point of four effectively differentiated the suicidal group from the control group with adequate sensitivity and specificity. The SSQ-OR total score was prospectively associated with subsequent suicidal ideation, plan, and attempt during the 6 months follow up, as were the sub-factor scores after adjusting for gender and age. Among the sub-factors, the odds ratio of social and environmental stress factor was consistently higher than suicide and mental health factor. These results suggest that stress is an important variable in suicide risk as well as vulnerability, as seen in the traditional stress-vulnerability model of psychopathology. Indeed, suicide researchers have proposed and proved stress-vulnerability models in suicide (47–49). Taken together, the inclusion of social and environmental stress along with STBs seems to be an advantage in risk assessment.

With regards to gender difference on SSQ-OR total score, the result did not display any difference, which is similar to previous studies on risk assessment that total scores of clinician-rated C-SSRS and self-reported BSSI did not differ by gender (25, 50–53). Although various risk factors for suicide differed by gender (54), it is essential to point out that a complex interplay is usually found between risk factors (9, 47). For example, age was identified as a relevant moderator for gender differences in suicide (55). In most countries, suicide risk is highest in older males, and the risk for suicide attempts is highest in younger females (56, 57). Since suicide risk is affected by the interaction of various factors, the difference in total score might not be noticeable simply by gender.

This study should be seen in the light of its strengths and limitations. To our knowledge, the SSQ-OR is the first observer rating scale that supplements the suicide risk assessment tools consisting of self-reports or clinician interview scales. We developed a comprehensive screening scale that includes a broader context of risk factors as well as STBs. In particular, because warning signs are included in the scale, we intended to detect observable behaviors that signal the imminent risk of STBs. Limitations include the difficulties associated with the possibility of systematic distortion in the rater's observations (e.g., Rosenthal effect, Halo effect). Moreover, it was reported that the level of agreement between self-report and observer varied according to the type of disturbance or the degree of change over time (58). Also, the floor effect was observed, which means that it could be hard to differentiate among the many individuals at a low level for SSQ-OR. Taking these points together, multi methodological assessment in combination with self-report and observer rating scale offers the best in identifying suicide risk. It is important to use the observer rating with a self-report or clinician-administered scale rather than using the SSQ-OR alone. Clinicians could also refine risk formulation by exploring potential areas of discrepancy between the information reported in the SSQ-OR and during the clinical interview (59). Second, although nationwide multi-site sampling was performed, it was validated only for the East Asian population. Future studies in various cultures are necessary to generalize these findings. Finally, due to the limitation of the study design, a 6 months follow-up was conducted for only a subsample of the participants recruited from the Crisis Response Centers.

In conclusion, the results of this study indicate the SSQ-OR appears to be a promising screening measure of suicidal risk. The SSQ-OR exhibits sound psychometric properties with good reliability, validity, and screening ability. The SSQ-OR is a useful screening tool to manage high-risk suicidal individuals in schools, community centers, or clinical settings. The SSQ-OR could be used as a complement to a self-report or clinical-administered scale to screen suicide risk comprehensively. Clinicians and researchers should carefully consider the strengths and weaknesses of this tool before employing it.

## Data availability statement

The raw data supporting the conclusions of this article will be made available by the authors, without undue reservation.

## Ethics statement

The studies involving human participants were reviewed and approved by Institutional Review Board of Samsung Medical Center. The patients/participants provided their written informed consent to participate in this study.

## Author contributions

Y-HC contributed to the search for background literature, to conduct the statistical analysis, to writing the original draft of the manuscript, to reviewing, and to editing the subsequent manuscript revisions. VY did the study design, coordinated the study, participated in statistical analysis, and provided discussion of results. KY and YC interpreted the data and discussed the study results. DeL coordinated the study, participated in statistical analysis, and interpreted the data. HJL coordinated the study and discussed the study results. DoL did the study design, coordinated the study, interpreted the data, and discussed the study results. HJJ contributed to conceptualization, project administration, and supervision. All authors contributed to writing and editing the manuscript.

## Funding

This work was supported by the Technology Innovation Program (or Industrial Strategic Technology Development Program-Source Technology Development and Commercialization of Digital Therapeutics) (20014967, Development of Digital Therapeutics for Depression from COVID19) funded by the Ministry of Trade, Industry and Energy (MOTIE, Korea), and by a grant of the Korea Health Technology R&D Project through the Korea Health Industry Development Institute (KHIDI) funded by the Ministry of Health and Welfare, Republic of Korea (HR21C0885).

## Conflict of interest

The authors declare that the research was conducted in the absence of any commercial or financial relationships that could be construed as a potential conflict of interest.

## Publisher's note

All claims expressed in this article are solely those of the authors and do not necessarily represent those of their affiliated organizations, or those of the publisher, the editors and the reviewers. Any product that may be evaluated in this article, or claim that may be made by its manufacturer, is not guaranteed or endorsed by the publisher.
