# Supplementary material for: Development and validation study of the suicide screening questionnaire-observer rating (SSQ-OR)
Source: Front Psychiatry. 2022 Aug 12;13:945051. doi: 10.3389/fpsyt.2022.945051 (PMC9411983; doi:10.3389/fpsyt.2022.945051)
Supplement: Supplementary file 1 [file Data_Sheet_1.docx]

# Appendix A.

# *Initial and final items for Suicide Screening Questionnaire-Observer Rating (SSQ-OR), with ratings for adequacy and importance of each item.*

| Pilot | Final | Content | A^a^ | I^a^ | Pilot | Final | Content | A^a^ | I^a^ |
| --- | --- | --- | --- | --- | --- | --- | --- | --- | --- |
| 1 | 4 | Depreciates or regards him/herself as pathetic. ^*^ | 3.82 | 3.71 | 21 |  | Rarely eats or binge eats. | 3.65 | 3.53 |
| 2 | 5 | Impulsively does something dangerous or regrettable. | 3.82 | 3.59 | 22 | 19 | Is being suspicious of other people’s intention and/or thinks others are doing him/her harm. | 3.24 | 3.47 |
| 3 | 6 | Says that he/she will die when emotions get intense. | 3.76 | 3.88 | 23 | 9 | Says that death is the only way to solve the current problems. | 4.47 | 4.47 |
| 4 |  | Sudden anger or aggressive behavior over trivial matters. | 3.71 | 3.88 | 24 | 12 | Says that people around him/her would be better off if he/she dies or disappears. | 4.35 | 4.29 |
| 5 | 10 | Talks about suicide or death. ^*^ | 4.82 | 4.94 | 25 |  | Says that even if the/she die, no one will care. | 3.88 | 4.00 |
| 6 | 8 | Has attempted suicide more than once so far. ^*^ | 4.71 | 4.41 | 26 |  | Has an envy or a tendency to imitate news of another person's suicide. | 3.82 | 3.65 |
| 7 |  | Has attempted suicide within the past year. | 4.65 | 4.29 | 27 |  | Speaks or threatens subject to revenge by suicide.. | 3.71 | 3.82 |
| 8 | 7 | Has planned a place, time, method for suicide. | 4.53 | 4.24 | 28 | 21 | Suffers from the financial distress. | 4.47 | 4.24 |
| 9 |  | Says that he/she wanted to die following a deceased family member, friend, pet or celebrity. | 4.47 | 4.35 | 29 | 1 | Rarely meets anyone and spends most of the day alone. | 4.41 | 4.29 |
| 10 | 13 | Has searched the Internet about suicide and/or has written online post/message implying suicide. | 4.41 | 4.47 | 30 | 24 | Suffers from being unemployed. | 4.35 | 4.35 |
| 11 |  | Says he/she understood the feelings of those who have committed suicide. | 4.24 | 4.24 | 31 |  | Suffers from the death of a loved one. | 4.29 | 4.41 |
| 12 | 11 | Suddenly organizes the surroundings. | 4.24 | 4.18 | 32 |  | Suffers from a failure. | 4.29 | 4.29 |
| 13 | 15 | Has hurt him/herself to the extent of leaving a scar. | 3.76 | 3.76 | 33 | 22 | Suffers from an illness or physical pain. ^*^ | 4.24 | 4.35 |
| 14 |  | Says he/she wasn’t afraid to die. | 3.71 | 3.88 | 34 |  | Does not come to social gatherings and receive calls well. | 4.18 | 4.12 |
| 15 |  | Complains extreme depression. | 4.47 | 4.18 | 35 | 3 | Suffers from conflict, divorce, or breakup with a lover or spouse. | 4.12 | 4.00 |
| 16 | 16 | Continues drinking even though drinking causes serious problems. | 4.24 | 4.12 | 36 | 2 | Suffers from conflicts with family members such as parents, children, or siblings. | 4.06 | 4.18 |
| 17 | 14 | Looks depressed or lethargic almost every day. ^*^ | 4.12 | 4.24 | 37 | 23 | Has suffered since experiencing physical, verbal or sexual violence. | 4.06 | 3.94 |
| 18 | 17 | Looks very anxious and nervous. | 3.94 | 4.00 | 38 | 25 | Suffers from unfair treatment or insult at work. | 3.76 | 3.88 |
| 19 | 20 | Complains about sleep problems. ^*^ | 3.88 | 3.65 | 39 |  | Has a physical disability that interferes with daily life. | 3.06 | 3.24 |
| 20 | 18 | Has severe mood swings. | 3.71 | 3.88 |  |  |  |  |  |

^a^ A = Adequacy of the item; I = Importance of the item, ^*^ Warning signs observed in more than 30% of suicide deaths in psychological autopsy results.

# Appendix B.

*Mean, Standard Deviation, and Item-Total Correlation of Suicide Screening Questionnaire-Observer Rating (SSQ-OR).*

| Item | *M* | *SD* | $r_{\mathrm{it}}$ | α | % |
| --- | --- | --- | --- | --- | --- |
| 1 | 3.875 | 23.086 | 0.380 | 0.904 | 1.40% |
| 2 | 3.868 | 22.630 | 0.487 | 0.902 | 4.20% |
| 3 | 4.043 | 23.851 | 0.322 | 0.904 | 1.40% |
| 4 | 3.833 | 21.942 | 0.633 | 0.898 | 2.10% |
| 5 | 3.979 | 22.872 | 0.528 | 0.900 | 1.86% |
| 6 | 4.016 | 22.856 | 0.597 | 0.899 | 3.15% |
| 7 | 4.012 | 22.828 | 0.596 | 0.899 | 4.20% |
| 8 | 3.968 | 22.527 | 0.612 | 0.898 | 6.18% |
| 9 | 4.049 | 23.238 | 0.547 | 0.900 | 2.68% |
| 10 | 3.944 | 22.284 | 0.646 | 0.898 | 1.28% |
| 11 | 4.095 | 23.886 | 0.439 | 0.903 | 2.10% |
| 12 | 4.061 | 23.344 | 0.541 | 0.901 | 2.56% |
| 13 | 4.052 | 23.242 | 0.552 | 0.900 | 6.41% |
| 14 | 3.918 | 22.104 | 0.663 | 0.897 | 2.45% |
| 15 | 4.029 | 23.363 | 0.459 | 0.902 | 3.26% |
| 16 | 4.099 | 24.256 | 0.281 | 0.905 | 2.68% |
| 17 | 3.962 | 22.533 | 0.602 | 0.899 | 1.98% |
| 18 | 3.864 | 22.153 | 0.601 | 0.899 | 4.31% |
| 19 | 3.982 | 22.980 | 0.502 | 0.901 | 2.68% |
| 20 | 3.766 | 22.217 | 0.537 | 0.901 | 3.73% |
| 21 | 3.982 | 23.069 | 0.476 | 0.901 | 4.43% |
| 22 | 4.046 | 23.879 | 0.316 | 0.904 | 1.40% |
| 23 | 4.068 | 23.678 | 0.436 | 0.902 | 2.10% |
| 24 | 4.083 | 24.004 | 0.348 | 0.904 | 0.93% |
| 25 | 4.013 | 23.585 | 0.363 | 0.904 | 3.50% |

M = scale mean if item deleted, SD = scale variance if item deleted, $r_{it}$ = corrected item-total correlations, α = Cronbach's Alpha if item deleted, % = percentage of responding to ‘do not know’.

# Appendix C.

*Eigenvalues and proportion of explained variance.*

| Variable | Eigenvalue | Difference between Eigenvalue | Proportion of common variance | Cumulative proportion of variance |
| --- | --- | --- | --- | --- |
| 1 | 7.67 | 6.29 | 0.46 | 0.46 |
| 2 | 1.37 | 0.30 | 0.08 | 0.55 |
| 3 | 1.07 | 0.26 | 0.06 |  |
| 4 | 0.81 | 0.03 | 0.05 |  |
| 5 | 0.78 | 0.10 | 0.05 |  |
| 6 | 0.68 | 0.10 | 0.04 |  |
| 7 | 0.58 | 0.05 | 0.03 |  |
| 8 | 0.53 | 0.08 | 0.03 |  |
| 9 | 0.45 | 0.08 | 0.03 |  |
| 10 | 0.38 | 0.38 | 0.02 |  |
| ︙ | ︙ | ︙ | ︙ |  |

Total common variance = 16.587.

# Appendix D.

*Parallel Analysis Obtained by Minimum Rank Factor Analysis.*

| Numbers of Factors | Real-data Eigenvalue | Random Data Eigenvalue  (95^th^ Percentile) |
| --- | --- | --- |
| 1 | 36.55* | 8.81 |
| 2 | 7.58 | 8.20 |
| 3 | 5.84 | 7.81 |
| 4 | 4.96 | 7.34 |
| 5 | 4.86 | 6.96 |
| 6 | 3.89 | 6.60 |
| 7 | 3.71 | 6.22 |
| 8 | 3.40 | 5.88 |
| 9 | 3.32 | 5.55 |
| 10 | 3.10 | 5.21 |
| ︙ | ︙ | ︙ |

* Advised number of dimensions.

# Appendix E.

*Factor Loadings of SSQ-OR from factor analysis using geomin rotation.*

|  | EFA ( *N* = 505 ) | | CFA ( *N* = 484 ) | |
| --- | --- | --- | --- | --- |
| Item | Factor 1 | Factor 2 | Factor 1 | Factor 2 |
| 1 | **0.773** | -0.184 | **0.510** |  |
| 2 | 0.058 | **0.718** |  | **0.689** |
| 3 | 0.057 | **0.570** |  | **0.532** |
| 4 | **0.780** | 0.089 | **0.844** |  |
| 5 | **0.635** | 0.134 | **0.739** |  |
| 6 | **0.520** | 0.345 | **0.89** |  |
| 7 | **0.768** | 0.089 | **0.853** |  |
| 8 | **0.891** | -0.036 | **0.829** |  |
| 9 | **0.792** | 0.118 | **0.811** |  |
| 10 | 0.398 | **0.548** |  | **0.932** |
| 11 | **1.027** | -0.306 | **0.83** |  |
| 12 | **0.867** | 0.032 | **0.796** |  |
| 13 | **0.799** | 0.003 | **0.86** |  |
| 14 | **0.903** | -0.002 | **0.858** |  |
| 15 | **0.933** | -0.252 | **0.711** |  |
| 16 | 0.132 | **0.561** |  | **0.551** |
| 17 | **0.852** | 0.021 | **0.782** |  |
| 18 | **0.601** | 0.244 | **0.809** |  |
| 19 | 0.283 | 0.**476** |  | **0.787** |
| 20 | **0.687** | 0.139 | **0.716** |  |
| 21 | 0.243 | **0.548** |  | **0.67** |
| 22 | 0.105 | **0.426** |  | **0.583** |
| 23 | 0.128 | **0.737** |  | **0.697** |
| 24 | -0.002 | **0.733** |  | **0.624** |
| 25 | -0.247 | **0.951** |  | **0.582** |

Factor loadings greater than .40 are in bold; EFA = exploratory factor analysis, CFA = confirmatory factor analysis, Factor 1 = Suicide and mental health; Factor 2 = Social and environmental stress.
